# Who is excluded and how? An analysis of community spaces for maternal and child health in Pakistan

**DOI:** 10.1186/s12961-015-0043-6

**Published:** 2015-11-25

**Authors:** Ayesha Aziz, Fazal Ali Khan, Geof Wood

**Affiliations:** Research and Knowledge Management Section, Rural Support Programmes Network, Islamabad, 44,000 Pakistan; International Development, University of Bath, Bath, UK

**Keywords:** Community spaces, Health, Maternal and child health, Social exclusion

## Abstract

**Background:**

The maternal, newborn, and child health (MNCH) indicators of Pakistan depict the deplorable state of the poor and rural women and children. Many MNCH programmes stress the need to engage the poor in community spaces. However, caste and class based hierarchies and gendered social norms exclude the lower caste poor women from accessing healthcare. To find pathways for improving the lives of the excluded, this study considers the social system as a whole and describes the mechanisms of exclusion in the externally created formal community spaces and their interaction with the indigenous informal spaces.

**Methods:**

The study used a qualitative case study design to identify the formal and informal community spaces in three purposively selected villages of Thatta, Rajanpur, and Ghizer districts. Community perspectives were gathered by conducting 37 focus group discussions, based on participatory rural appraisal tools, with separate groups of women and men. Relevant documents of six MNCH programmes were reviewed and 25 key informant interviews were conducted with programme staff.

**Results:**

We found that lower caste poor tenants and nomadic peasants were excluded from formal and informal spaces. The formal community spaces formed by MNCH programmes across Pakistan included fixed, small transitory, large transitory, and emerging institutional spaces. Programme guidelines mandated selection of community notables in groups/committees and used criteria that prevented registration of nomadic groups as eligible clients. The selection criteria and adverse attitude of healthcare workers, along with inadequacy of programmatic resources to sustain outreach activities also contributed to exclusion of the lower caste poor women from formal spaces. The informal community spaces were mostly gender segregated. Infrequently, MNCH information trickled down from the better-off to the lower caste poor women through transitory interactions in the informal domestic sphere.

**Conclusion:**

A revision of the purpose and implementation mechanisms for MNCH programmes is mandated to transform formal health spaces into sites of equitable healthcare.

## Background

Maternal, newborn, and child mortality remain high in most parts of Pakistan. With a neonatal mortality rate of 55 per 1,000 live births [1], Pakistan is one of the 10 countries contributing to 67% of neonatal deaths worldwide [2]. With a maternal mortality ratio of 276 per 100,000 live births [1], Pakistan ranks third among the six countries accounting for more than 50% of global maternal deaths [3].

Maternal, newborn, and child health (MNCH) indicators of Pakistan depict wide disparities as neonatal mortality is about 28% higher for the poorest 20% households as compared to the richest 20% [1]; only 27% of women in the lowest wealth quintile deliver in a health facility as opposed to 84% of women in the highest wealth quintile. Maternal and child healthcare is one of the most inequitably distributed health resources [4]. Disparities in access to healthcare services are known to be influenced by sociocultural hierarchies that marginalise particular groups [5].

Poverty, rural residence, and lack of education are well-known risk factors for maternal and child mortality [1]. However, access to healthcare services is a far more complex issue mediated by intertwined identities of caste, class, and gender, particularly for the women and children living at economic and social margins [6].

In Pakistan, caste or ‘zaat’, and ethnic identities are interlinked with land ownership, occupation, and access to and control over resources [7]. They are the primary determinants of hierarchical relationships that result in social exclusion and economic and political exploitation of the lower castes in Pakistan [8, 9]. Ethnic and class based polarisation has prevented human development in the country [10]. Religion has been found to be a source of stratification linked with lineage in some areas of the country, but gender division of space and power cuts across all other forms of social marginalisation [8, 11]. Women’s status and mobility in public spaces is restricted by familial and kinship boundaries that limit their access to and utilisation of healthcare [12, 13].

Pakistan’s National Maternal and Child Health Policy and Strategic Framework, 2005–2015, is committed to addressing inequities in access to and utilisation of MNCH services [14]. Government and non-government programmes have focused on promoting safe motherhood practices, such as skilled birth attendance, family planning, and emergency obstetric care [15–17]. However, Pakistan is far from meeting the targets set for Millennium Development Goals 4 and 5. Both supply and demand side interventions for maternal and child health have achieved limited success as they fail to recognise the multidimensional nature of poverty and the lived realities of poor, low caste women [6]. Recognition of health systems as core social institutions is imperative to address the systemic inequities and cultural marginalisation that adversely affect the lives of women and children [18]. We suggest that application of the concept of community spaces can be used to identify structural mechanisms and social determinants of exclusion that control access to MNCH services.

Space is a relational concept that shapes human behaviour and is construed by the position and relation of people, objects, and events occupying it [19]. It may be considered akin to a physical location, an area with social dimensions, or an abstract mental frame [20]. Design of community spaces has been associated with the politics of colonisation [20–22]. Bourdieu [23] argued that participation in social networks and mobilisation of resources by people (social actors) generates social capital and domination. Space is denoted by terms like environment, surroundings, and territory that are settings in which social activities occur [24]. In health, the term ‘setting’ has been used to describe locations where people interact and shape their environment and as a result create or solve health-related problems [25]. While the term ‘setting’ implies formal structure, its definition connotes the structural and sociological effects of spaces on human behaviour and the impact of human identities and relationships on the shape of spaces [26]. The interactive relationship between community spaces and human behaviour remains largely unacknowledged in health literature.

Recently, a study to understand why and how resources are unequally distributed between groups of people has applied the social relations lens to make sense of social exclusion in maternal and child health systems [27]. However, participation in social networks and physical and social positioning of poor, lower caste individuals and groups in private and public spaces needs to be viewed with respect to their security and risk management strategies [28]. This study locates the notion of social exclusion in the physical and social dimensions of community spaces, which are fundamental for communal life and exercise of power [29]. Empowering participation has been recognised to have a positive impact on the health of disadvantaged groups by reducing social exclusion and improving access to healthcare [30]. Many MNCH programmes in Pakistan aim to engage local people in health groups, committees, or community support organisations. However, externally administered development has been found to reinforce inequities rooted in society [31]. Social segregation and disadvantage transcends across the private sphere of households and the public sphere of indigenous communal spaces [32]. To find pathways for improving the lives of the excluded, the social system of community spaces needs to be treated as a whole so that problems of participation at all levels can be unearthed.

Gaventa [33] distinguished the ‘provided or closed’ spaces controlled by the elite groups and the ‘invited’ spaces created by institutions and policymakers from the ‘claimed’ spaces constructed by community people. He proposed that ‘places of engagement’ existed within one or more spaces where different forms of power (visible, hidden, and invisible) operated in the form of inclusion or marginalisation [33]. This study has adapted Gaventa’s model to categorise community spaces into formal ones, those created or facilitated by MNCH programmes, and informal ones, which are indigenous places for women and/or men where they get together and discuss social, economic, and health issues. After a presentation of social structure in the study villages, it identifies the different types of community spaces and describes the modes of exclusion operating in them to suggest directions for design of more inclusive and equitable MNCH programme strategies.

## Methods

The study was conducted between April 2013 and March 2014 using a qualitative case study design in three purposively selected villages across Pakistan.

Pakistan is administratively divided into the four provinces of Sindh, Punjab, Balochistan, and Khyber Pakhton khwa and the two regions of Gilgit-Baltistan and Azad Jammu and Kashmir. Considering that the geographic terrain of the country includes mountainous areas, plains, desserts, and the delta region, three diverse districts, Thatta in Sindh, Rajanpur in Punjab, and Ghizer in Gilgit-Baltistan, were purposively selected for the study. Vulnerability ranking and ease of access were important considerations during selection of the districts. In consultation with the local MNCH programme implementers, one village in each district and its inhabitants were chosen to be studied as a case. Selection of villages where at least three MNCH programmes were working and willing to cooperate was important. In Thatta, Jani Memon village was selected; Basti Basheer Nagar in Rajanpur and Sumal village in Ghizer were the study sites in the other districts. The village case studies served as ‘keyhole’ observations for identifying individuals and/or groups that were excluded from community spaces and understanding the mechanisms for their exclusion.

Data about formal and informal community spaces in each of the three villages was collected through observations and focus group discussions (FGDs) with community women and men. FGDs were based on participatory rural appraisal tools, which use symbols, figures, and pictorials to enable even the illiterate rural people to become analysts of their own realities and facilitate them to take collective actions for improvement [34]. The participatory rural appraisal tools used during FGDs of this study included transect walks (purposeful systematic walks across the study site), social maps, network diagrams, and cause and effect diagrams. For building trust and rapport with the villagers, a field team comprising of five members (a male or female field supervisor, two female and two male field researchers with background knowledge of social sciences and familiarity with local culture and languages) roamed around the area, visited households, and met different people in each village. Consent to initiate field inquiry was obtained from the villagers and MNCH programme staff.

After developing an understanding of the spatial boundaries and positioning of different castes in the study villages, the field team asked some women and men from each group to accompany them in conducting a transect walk. Discussions and observations about communal places and activities, and people’s participation in them helped the field team in identifying the different castes and socially excluded groups in each village. Women and men from all castes and socioeconomic strata were invited to participate in group discussions that began with a social mapping exercise. As women and men made social maps of their village they identified various formal spaces created by the MNCH programmes and the informal spaces for women and men. Villagers told the field team about who was able to go where and why. The network diagram facilitated understanding of bonds between people, within and across their castes, and with the community- or facility-based healthcare staff. People described how groups or committees were formed by MNCH programmes and related experiences about how they were treated. The cause and effect diagram helped in comprehending the contributions of the formal community spaces, particularly with respect to raising awareness, promoting community mobilisation, and improving local accountability.

Data on formal spaces was triangulated with information about MNCH programmes through review of relevant documents and conducting key informant interviews (KIIs) with programmatic staff. The KIIs were based on semi-structured guidelines and along with the document review they facilitated understanding of programmatic guidelines for formation of formal spaces and their criteria of inclusion and exclusion. They also allowed comprehension of programmatic aims with respect to community mobilisation.

The research tools were pilot tested, revised, and compiled into a manual for field work [35] after approval by the Ethics Review Committee of the Research and Advocacy Fund (the funding agency). The field teams underwent a 10-day training based on the concepts of the study and familiarised themselves with the field entry guidelines and research tools in the manual. During field work the field teams were supervised by the Principal Investigator (FAK) and the Co-Investigator (AA).

A total of 25 KIIs of 1–1.5 hour duration each, were conducted with programme management and field staff of six MNCH programmes working in the three selected villages. The names of the selected villages and the MNCH programmes studied in them are shown in Table 1.

**Table 1:** Name of selected research cites and MNCH programmes

**District**	**Village**	**MNCH programmes**
Thatta	Jani Memon	The National Programme for Family Planning and Primary Healthcare
		The Population Welfare Programme
		The Maternal and Child Health Programme of Merlin
Rajanpur	Basti Basheer Nagar	The National Programme for Family Planning and Primary Healthcare
		The Population Welfare Programme
		The Maternal and Child Nutrition Programme of the Lodhran Pilot Project
Ghizer	Sumal	The National Programme for Family Planning and Primary Healthcare
		The Population Welfare Programme
		The Maternal, Neonatal and Child Health Programme
		The Aga Khan Health Services Pakistan

The FGDs were conducted with a total of 120 participants (59 females and 61 males). Table 2 shows the characteristics of research participants in each study village with respect to age groups, gender, and educational and economic status. The initial plan of conducting 10 FGDs of 2 hours long in each village was revised to conduct a total of 37 FGDs of 1.5–2 hours each with separate groups of women and men because participants were not able to stay for the complete duration. Both the women’s groups and the men’s groups consisted of 15–30 participants. The principle of selection was to capture diversity with respect to inclusion and socioeconomic status and not representativeness.

**Table 2:** Demographic and socioeconomic characteristics of research participants

**Characteristics**	**Districts**			**Total**
	**Thatta**	**Rajanpur**	**Ghizer**	
	**(n = 30)**	**(n = 55)**	**(n = 35)**	**(n = 120)**
Sex				
Female	15	28	16	59
Male	15	27	19	61
Age group, years				
16–30	13	15	12	40
31–45	11	30	15	56
46–60	4	10	5	19
>60	2	0	3	5
Educational status				
Not literate	17	39	0	56
Primary	9	5	3	17
Higher secondary	3	9	30	42
Above higher secondary	1	2	2	5
Marital status				
Unmarried	7	2	2	11
Married	23	53	33	109
Economic status				
Very poor (nomads and landless peasants)	9	15	5	29
Poor (tenants and small land owners)	11	27	19	57
Better-off (big land and business owners)	10	13	11	34

Data from the KIIs and FGDs was gathered in local languages and recorded as audio files. The audio files were transcribed and translated into field notes that were entered in a computerised format. Measures to monitor quality and ensure confidentiality of data were taken at all steps. Using NVivo 10.0 version, the computerised data was coded with respect to study site and categorisation of community spaces. Themes and sub-themes were generated to identify the excluded and the mechanisms for their exclusion in the three study villages. The emerging themes were carefully considered during collective deliberation meetings of the research team. Similarities and differences between study sites were used to merge data into common and atypical themes.

## Results

### Social structure of study villages

Stratification in each study village was constructed by a combination of various social and economic identities. Caste was the main source of social stratification in Thatta and Rajanpur, while religious sect was the primary source of social segregation in Ghizer. Caste and religious sect based identities overlapped with other forms of socioeconomic division. People belonging to the upper castes and the dominant religious sect were usually better-off not only in economic status, but also with respect to education, housing infrastructure, and access to transport and healthcare resources. People of the other castes or sects were poor tenants or small land owners and the very poor nomads or landless peasants.

In Thatta and Rajanpur, people from the same caste were usually relatives. They lived close to each other in small settlements and marriages rarely took place outside the castes. Village Jani Memon in District Thatta was inhabited by the Memons (the landowner caste or ‘zaat’), the Khaskhelis (the tenants and working caste), and the Shaikhs (the daily wage caste). The Punjabi and Sindhi Arain castes (biraderis) were the big landowners in Basti Basheer Nagar of District Rajanpur, while the Jhangars and Ghauris owned small pieces of land or lived as tenants. People from other castes, including the Pathans, Utairas, and Baluchs, were nomadic peasants or daily wagers. The tenants lived on and cultivated the agricultural fields of the better-off. They also reared their livestock, as their livelihood depended on taking a small share (one-eighth) of the agricultural and animal products. The lower caste nomadic peasants moved from place to place in search of livelihood as they worked in agricultural fields on a daily wage basis or earned a daily living by selling harvests. Poor households in Thatta and Rajanpur used the agricultural fields for defecation and their women and children used muddy swamp water for washing. The women fetched drinking water 2–3 times a day from communal hand pumps. The government and private healthcare facilities were at a distance of almost 4 km from the selected villages in Thatta and Rajanpur. Most poor women faced economic and cultural constraints in accessing them as they had no means of transportation or the money to pay the service fee and buy prescribed medicines. Reliance on traditional remedies and home-based deliveries increased risk to the lives of poor women and children.

In Sumal village of District Ghizer, ethnic identities were derived from lineage, migration and settlement patterns, and linguistic differences. The dominant Ismaili religious sect included the Syeds, Sheen, Shairkhanay, and Gulchiniot castes, while the Rajas and Gujjars belonged to the minority Sunni sect. The Gujjars were recognised as the nomadic caste, who usually lived in the higher pastures where they grazed and reared the livestock of the better-off castes such as Syeds and Rajas. Except the Gujjars, people from all other castes owned small pieces of land. However, the Ismaili people were better-off as they had gained education and acquired jobs that increased their household income. A non-government women’s health centre provided subsidized antenatal and postnatal care and conducted facility-based deliveries. Since the health centre was located in the middle of Sumal village, the nomadic Gujjars faced difficulties in accessing it. Village-based healthcare workers also belonged to the Ismaili sect; therefore, the women from the Sunni sect felt inhibited in communicating with them.

### Types of formal and informal community spaces

The formal community spaces formed by the MNCH programmes working in the study villages included the four following types: (1) fixed spaces that were static as they existed in healthcare facilities and were formed during consultation/interaction between healthcare providers and visiting clients, who were usually women of reproductive age; (2) small transitory spaces that were not fixed in a particular place, but were formed during door-to-door visits of healthcare workers as they interacted with clients in their homes; (3) large transitory spaces that were formed when awareness raising sessions were conducted by healthcare workers. The place for these spaces was selected within the community settings and included courtyards of a big house or some public space like a school building. The healthcare workers usually contacted a known focal person (female and/or male) in the village and asked them to gather participants for the awareness session. The audience of these spaces usually consisted of women of reproductive age, though infrequently separate sessions were held with groups of adolescents or men; and (4) emerging institutional spaces that were created during group meetings of health groups or committees by the healthcare workers. The healthcare workers usually contacted the active women, healthcare workers of other programmes, and notable community men (e.g. big land owners, teachers, elected representatives) and formed a group. Then they convened the group meetings in community settings like courtyards of a big house or some public space like a health dispensary or school building. Mostly, separate groups of women and men were organized by female and male facilitators of MNCH programmes, but sometimes health groups or committees had both female and male members.

The informal community spaces identified in the private sphere included households, neighbours, kitchens gardens, and event celebrations. The informal public community spaces for men included the roadside, shops, shade of old trees, community guest house, schools, mosques, *Jamat Khanas* (the religious and community centre of the Ismaili sect in Ghizer district), religious shrines, and graveyards. Women occupied informal public spaces only transiently as they gathered around communal hand pumps and water channels to collect drinking water and wash clothes. Sometimes women also went to the woods to graze their animals or to the ladies shop that existed only in Ghizer district. Agricultural fields and markets were the only public spaces where men and women could be seen together.

### Mechanisms of exclusion in formal community spaces

Exclusion from the formal community spaces was mainly due to MNCH programme design and implementation strategies. MNCH programme guidelines mandated the inclusion of community notables, literate people, and healthcare workers in the large transitory and emerging institutional spaces, while no such specifications were made for inclusion of the lower castes that comprised of tenants, small land owners, nomadic peasants, and daily wage earners. Commenting on the process of group formation a programme staff member said that:

“*We include the people who are socially active and the women who can talk easily…people listen to the local leader* [Lumberdar] *so we have included him too.*”

The structure and function of these types of spaces was highly questionable as we found that awareness sessions were held irregularly, the structure of all community-based groups/committees was not very well-defined, and the roles and responsibilities of members were not clear. None of the awareness sessions or group meetings were found to inform community people of their rights and entitlements or help them in making collective and organised efforts for change. Contrarily, inclusion of community notables in formal spaces to influence behaviour change among the others reinforced the caste and class based power structures.

We also found that, in the fixed and small transitory spaces, people were registered as clients only if they had been living in the catchment area for more than 6 months; therefore, the nomadic population groups were excluded and could not utilise the MNCH services. A programme staff member elaborated:

“*According to our criteria we cannot register people unless they have been living in an area for more than six months. If they settle in a place, then we register them. If we have achieved our target…then we leave them. In case our target is not achieved till that time and the house of tenants is in our area, then we register them.*”

The community-based facilitators of MNCH programmes were selected on the basis of their educational status and therefore belonged to the better-off castes. In the formal spaces, these facilitators preferred to associate with their own relatives and friends as they continued to carry their prejudices against the lower caste poor groups. Some healthcare workers communicated in a language that the lower caste poor could not understand, while others maltreated them. The community - based cadres of healthcare workers were relatively more aware of the power dynamics existing in informal spaces as they discussed adverse effects on the health of poor women and children. However, they did little to benefit the poor, especially in terms of giving feedback and suggestions for improving programme strategies.

Lack of programmatic funding and resources also limited the geographic scope of activities, as outreach activities in poor and remote areas were conducted for short time periods, only when the programmes had adequate financial resources. Some MNCH programme staff said that, when they organized community-based outreach activities, they faced difficulties in including the poor and marginalised people in formal spaces as they were heavily engaged in livelihood activities.

The lower caste poor people also excluded themselves as they gave less value and attention to participation in formal spaces and their women’s health. Most of them could not afford to follow the instruction of healthcare workers and seemed resistant to adopting healthy behaviours, while others were restricted by social norms evident in the story of a poor low caste woman, as stated:

“*My in-laws live as a joint family… My husband and brothers-in-law are tenants and work for one eighth part of the total produce. So far, I have had six pregnancies, three of which resulted in birth of weak and jaundiced babies who died a few weeks after birth. Currently, I am in the ninth month of my seventh pregnancy. In our family women are not allowed to go outside alone so I only go to death and marriage ceremonies in the village, with the permission of my husband and my husband buys clothes and household goods for us. Our children do not go to school but contribute in earning as they start working in the fields at the age of five to seven. I never visited any hospital for vaccination of children, but once some team came to my house and gave vaccination to my children. I do not use any family planning method for birth spacing, except one time before the recent pregnancy. I took contraceptive tablets from the lady health worker, but the tablets reacted on me and I started to bleed severely so I never used the tablets again. Recently some women have told me about the major operation* [removal of uterus] *and I discussed it with my husband. He did not allow me to get the operation done because he says it is against Islam, but he agreed to use some other contraceptive method.*”

On the other hand, people from the better-off castes blamed the lower caste poor people for their choices by saying that:

“*They do not have good environment, they do not give importance to health and education. That is why they do not like to attend such* [health education] *programmes.*”

### Interaction of informal and formal community spaces

The informal spaces were mostly gender segregated and can be categorised into private and public spaces. Most public informal spaces were occupied by men (such as the mosques, shops, agricultural fields, *Jamat Khanas*, shade of old trees, and the roadsides) provided opportunities for interaction within and across caste and class boundaries. Therefore, men’s access to information and opportunities was far more than that of women whose interactions were usually limited within their own families and castes. Women’s claim over public informal spaces was infrequent and rather transitory. Women of all castes and classes had no restrictions in visiting the households of their relatives and neighbours who belonged to the same caste. However, only women from the poorer castes went further away from their households to wash clothes, fetch drinking water, and work in agricultural fields. Women’s solidarity was found to be limited by socioeconomic boundaries. The poorer women shared their problems openly and helped each other in resolving them. They helped in each other’s deliveries, arranging transportation, and accompanying each other to healthcare facilities. On the other hand, the social network of the women from the better-off castes was quite limited as they stayed in their own houses and usually interacted with their own family and friends. They felt low while interacting with lower caste poor women and did so only in some events and celebrations or when the poor women came to work in their households. Both the poor and better-off women told us that their men forbade them from developing very close relationships with each other. Spaces like *waranda* (open area alongside of the house), where events and celebrations in the private domain were held, and the religious shrines and *Jamat Khanas* in the public domain were open to women and men from all socioeconomic strata. Though the gatherings of women in these spaces were segregated from that of men, sometimes these were sites for information exchange related to MNCH issues as they were utilised by MNCH programmes to conduct separate health awareness sessions for women and men.

The networks formed and matters discussed in the informal spaces need special consideration as they affect social relationships and decisions related to MNCH issues. We found that discussions among men were usually focused on livelihood information and opportunities, while the women spoke to each other about the various issues affecting their lives, which included family disputes, economic problems, relationship with husband and in-laws, and even health problems. During their transitory interactions with the women from better-off castes, the lower caste poor women gained information about the dietary and healthcare needs of pregnant women and the necessity of birth spacing and using family planning services. This infiltration of information from the formal community spaces through the informal ones increased awareness and prompted behaviour change among the lower caste poor individuals. A collective movement for health and rights of the poor still seems to be a far sighted goal as it was not the agenda of any informal or formal community spaces.

## Discussion

Our study applied the concept of space to identify the structural mechanisms and social determinants of exclusion that adversely affect access to MNCH services. Our findings present a typology of formal community spaces which include fixed, small transitory, large transitory, and emerging institutional spaces created and/or facilitated by the MNCH programmes.

The poor, who usually belong to the lower castes and/or minority religious sect, are excluded from all the formal community spaces. Other studies have highlighted the economic and social exclusion of the poor and lower castes, particularly women across Pakistan [6, 8]. Caste based durable inequalities have also been found to impede progress towards achieving Millennium Development Goal targets in other South Asian countries [36]. Our study provides evidence of how this exclusion is sanctioned by the design and implementation guidelines of government and non-government MNCH programmes in Pakistan.

Our findings reveal that the guidelines for construction of formal health spaces emphasise inclusion of community notables and render the nomads as ineligible clients. The structure of formal health spaces maintains the influence and dominance of people from the better-off castes as their voice is valued for promoting behaviour change. Studies on participatory development and poverty reduction programmes have demonstrated similar reinforcement of power structures and subjugation of the poor, rural, and lower caste people [8, 31]. Analysis of maternal and child health related policies have acknowledged the necessity of equity objectives and research on addressing disparities in maternal healthcare has been undertaken [27, 37]. However, the critical question of the position of healthcare programmes in the larger sociopolitical space continues to be overlooked.

Our findings provide evidence of lack of programmatic interest in mobilising communities and raising awareness about their rights and entitlements. The irregular organisation of formal spaces, ambiguity in roles and responsibilities of their participants, and the absence of feedback from lower cadres of healthcare workers reflects the ignorance, incapacity, and apathy of MNCH programmes and their staff. Gaventa [33] argued that adoption of visible and hidden forms of power from the closed spaces into the invited ones is decided by the institutions and state. The crucial choice that maternal and child health and related development programmes in Pakistan will have to make is with respect to defining their core political position in relation to prevailing power dynamics. The objective of providing or increasing healthcare access of vulnerable people will require taking their side and redefining programme structures and strategies in their favour [38].

Besides underscoring the demand and supply side financial challenges in accessing healthcare, our study reveals the attitudinal barriers among healthcare workers and clients. We found that education of community-based MNCH programme facilitators was backed by their belongingness to the better-off castes, which prompted their dismissive and derogatory attitude towards the lower caste poor women. Reminded of their inferiority, the poor lower caste people excluded themselves from the formal spaces and seemed to give less value to their health. Structural poverty maintains poor people’s focus on secure livelihoods and obligates them to discount the future [28]. Voluntary exclusion is a function of restricted opportunities for participation or a response to discrimination [39]. The underlying systemic factors that increase vulnerability of the socially excluded must be carefully considered while designing MNCH programmes, their implementation, and accountability mechanisms [9].

Our findings illustrate gender based segregation of informal community spaces. Men from different castes and classes interacted frequently in public spaces, but livelihood was the primary reason for these bridging relationships [40]. Like Gazdar [12], we also found that women’s mobility was restricted within the boundaries of caste and kinship. The lower caste poor women had strong bonding capital as they helped each other with health emergencies, but their transient bridging ties with the better-off shows their limited access to health information. Our study could not measure the trickle down of health information through transient informal spaces, but has demonstrated its existence. Further research on measuring the trickle down effect of informal community spaces is necessary to weigh their potential. Saegert et al. [41] stressed upon the advantages of solidarity, but studies in the context of South Asia and Pakistan have highlighted the collective economic and relational disadvantages of poor communities [28, 42]. A paucity of state services mandates disadvantaged groups to seek protection through bridging social ties [9]. The inability to ensure reciprocity prompts adverse incorporation of the poor into a system that reproduces their poverty and disadvantage [28].

Our findings are not generalizable, but they present an analysis of the mechanisms of exclusion in formal and informal spaces in different geographic settings across Pakistan. Review and reform of programme objectives and implementation strategies is critical for addressing structural and social inequities related to maternal and child health. A step-wise approach to participatory empowerment of communities combined with an enabling environment of trained healthcare providers and accountability on equity measures is called for [31, 43].

## Conclusion

Female gender and membership of lower castes, poor class, or minority religious sects are determinants of social exclusion in formal and informal community spaces. The power dynamics of informal spaces keeps the poor lower caste women at the highest level of disadvantage. Health information trickles down to the poor lower caste women through transient bridging, informal social relations with the better-off. However, further research is required to explore the potential of informal community spaces. The formal community spaces formed by MNCH programmes across Pakistan include fixed, small transitory, large transitory, and emerging institutional spaces. Programme objectives, guidelines, eligibility criteria of clients, selection process, and attitude of healthcare workers are the key factors that must be revised to transform the formal spaces into sites of equitable healthcare.

## Abbreviations

FGDs: Focus group discussions
KIIs: Key informant interviews
MNCH: Maternal, newborn, and child health
